# Understanding interventions for improving routine immunization coverage in children in low- and middle-income countries: a systematic review protocol

**DOI:** 10.1186/2046-4053-2-106

**Published:** 2013-11-21

**Authors:** Shingai Machingaidze, Eva Rehfuess, Rüdiger von Kries, Gregory D Hussey, Charles S Wiysonge

**Affiliations:** 1 Vaccines for Africa Initiative (VACFA), Institute of Infectious Disease and Molecular Medicine (IIDMM), Health Sciences Faculty, University of Cape Town, Room N2.09A, Wernher + Beit North, Anzio Road, Observatory, 7925 Cape Town, South Africa; 2 Division of Medical Microbiology, Department of Clinical Laboratory Sciences, University of Cape Town, Cape Town, South Africa; 3 Center for International Health, University of Munich (LMU), Munich, Germany; 4 Institute for Medical Informatics, Biostatistics and Epidemiology, University of Munich (LMU), Munich, Germany; 5 Institute of Social Pediatrics and Adolescent Medicine, University of Munich (LMU), Munich, Germany; 6 Centre for Evidence-based Health Care, Faculty of Medicine and Health Sciences, Stellenbosch University, Tygerberg, 7505 Cape Town, South Africa

**Keywords:** Expanded Program on Immunization (EPI), Routine immunization, Routine vaccination, Children, Low- and middle-income countries

## Abstract

**Background:**

Virtually all low- and middle-income countries are dependent on the World Health Organization’s Expanded Program on Immunization for delivery of vaccines to children. The Expanded Program on Immunization delivers routine immunization services from health facilities free of charge. Understanding interventions for improving immunization coverage remains key in achieving universal childhood immunization.

**Methods:**

We will conduct a systematic review that aims to assess the effectiveness of the full range of potential interventions to improve routine immunization coverage in children in low- and middle-income countries. We will include intervention studies, as well as observational studies. We will search the Cochrane Database of Systematic Reviews (CDSR), Cochrane Central Register of Controlled Trials (CENTRAL), MEDLINE, EMBASE, electronic databases for eligible studies published by 31 August 2013. At least two authors will independently screen search outputs, select studies, extract data and assess the risk of bias (using separate criteria for interventions and observational studies); resolving any disagreements by discussion and consensus. The use of logic models and the Cochrane Complexity Matrix will be explored in order to better understand and contextualize studies. We will express the result of each study as a risk ratio with its corresponding 95% confidence intervals for dichotomous data, or mean difference with its standard deviation for continuous data. We will conduct meta-analysis for the same type of participants, interventions, study designs, and outcome measures where homogeneity of data allows. Use of harvest plots may be explored as an alternative. Heterogeneity will be assessed using the χ^2^ test of heterogeneity, and quantified using the I^2^ statistic. This protocol has not been registered with PROSPERO.

**Discussion:**

This review will allow us to document evidence across a broad range of intervention types for improving routine immunization coverage in children and also distinguish between those that are well supported by evidence (to direct policy recommendations) and those that are not well supported (to direct research agenda).

## Background

Following the successful eradication of smallpox, the World Health Organization (WHO) launched the Expanded Program on Immunization (EPI) in 1974 with the hope of achieving 80% coverage of children less than 1 year of age with vaccines against six major causes of death among children (measles, diphtheria, tetanus, polio, tuberculosis and pertussis) by 1990 [1,2]. Virtually all low- and middle-income countries (LMICs) are dependent on EPI for delivery of vaccines to children. EPI delivers routine immunization services from health facilities free of charge. ‘Routine immunization’ services rely on residents going to fixed sites to receive a service that is offered regularly throughout the year. Routine immunization services may also include mobile teams, which take services at regular intervals to populations without nearby health centers; and ‘outreach activities’ which reach out regularly to the community to provide a service or retrieve defaulters [3].

While routine immunization schedules may vary by country, the vaccines shown in Table 1 are recommended by WHO. Some vaccines such as those against measles, diphtheria, tetanus and pertussis are recommended for all children; others such as those against yellow fever and Japanese encephalitis are recommended only for children residing in areas where such diseases are endemic; and others such as cholera and typhoid vaccines are recommended for children in some high-risk populations. In addition, some vaccines are given as combination vaccines such as the pentavalent vaccine against diphtheria, tetanus, pertussis, hepatitis B (Hep B) and *Haemophilus influenzae* type b (Hib).

**Table 1:** **Vaccines recommended for children by the World Health Organization (WHO) typically given in low- and middle-income countries (LMICs)**^**a**^

**Vaccine**	**Doses**	**Age**	**Minimum interval**
BCG	1	Birth or soon after	Not applicable
OPV	4	Birth, 6, 10, 14 weeks	4 weeks
DTP	3	6, 10, 14 weeks	4 weeks
HepB^b^	3/4	Birth, 6, 10, 14 weeks	4 weeks
Hib	3	6, 10, 14 weeks	4 weeks
PCV	3	6, 10, 14 weeks	4 weeks
RV^c^	2/3	6, 10, 14 weeks	4 weeks
Measles	1	9 months	Not applicable
Rubella^d^	1	9 or 12 months	Not applicable
Yellow fever	1	9 months	Not applicable
Vitamin A	2	9, 15 months	6 months
HPV^e^	3	9 to 13 years	Variable
Meningococca^f^	1/2	>9 months	Variable
Japanese encephalitis^g^	1/2	9 to 12 months	4 weeks

^a^Full list of recommended vaccines here: http://www.who.int/immunization/policy/Immunization_routine_table2.pdf.

^b^Some countries have a policy of giving a birth dose of the hepatitis B vaccine.

^c^There are two types of licensed rotavirus vaccines, Rotateq and Rotarix. Rotarix is given in two doses, while a full series of Rotateq vaccination consists of three doses. The first dose of the rotavirus vaccine should be administered between 6 and 14 weeks. The maximum age for administering the last dose of the vaccine should be 32 weeks.

^d^Minimum age for giving rubella is 6 months.

^e^There are two types of licensed HPV vaccines. The quadrivalent vaccine is given between 9 to 13 years with a 4-week minimum interval between the first and second dose, and a minimum of 12 weeks between the second and third dose. The bivalent vaccine is given between 10 to 13 years with a maximum of 2.5 months between the first and second dose.

^f^There are three meningococcal vaccines available: Men A conjugate, one dose given between 1 and 29 years; Men C conjugate, two doses given between 2 and 11 months with a minimal interval of 8 weeks between first and second dose, or one dose given >12 years; quadrivalent conjugate, two doses between 9 and 23 months, with 12 weeks between the first and second dose, or one dose given at >2 years.

^g^There are two vaccines available: the mouse-brain-derived vaccine, two doses given at 1 year, with 4 weeks between first and second dose; and the live attenuated vaccine, one dose given between 9 and 12 months.

*BCG* Bacille Calmette-Guérin, *DTP* diphtheria-tetanus-pertussis vaccine, *HepB* hepatitis B vaccine, *Hib Haemophilus influenzae* type b vaccine, *HPV* human papilloma virus, *PCV* pneumococcal conjugate vaccine, *OPV* oral polio vaccine, *RV* rotavirus vaccine.

Coverage with three doses of the diphtheria-tetanus-pertussis vaccine (DTP3) by 1 year of age is widely accepted as a proxy for measuring overall EPI performance. In 2011, global DTP3 coverage reached 83%. However, Africa lagged behind and coverage reached only 74% [4]. Poor vaccination coverage in LMICs has been attributed to several reasons associated with immunization systems, parental attitude and knowledge, communication and information, and family characteristics [5-8]. Therefore, interventions for improving childhood immunization coverage may target parents and caregivers in the community, the service provider, the health system, or a unique combination of any of these. Interventions for improving childhood immunization coverage in LMICs have recently been assessed in a systematic review [9]. However, only randomized controlled trials (RCTs), non-randomized controlled trials (NRCTs) and interrupted time series (ITS) studies were eligible for inclusion in the review, and only six studies met the inclusion criteria. As not all health systems interventions lend themselves to being investigated through RCTs, it is important to look beyond these study designs of high internal validity to identify other interventions of potential relevance.

### Objectives

We aim to assess the effectiveness of the full range of potential interventions to improve routine immunization coverage in children in LMICs. The review will address the question of ‘which interventions work?’ and provide some insight towards ‘how, why, and for whom these interventions work?’.

## Methods

A logic model will be developed to help with scoping the review, defining and conducting the review and making the review relevant to policy and practice [10], using templates developed as part of the EU-funded INTEGRATE-HTA project (Anke Rohwer, personal communication).

We will also attempt to capture the complexity of the different interventions included in the review by assessing the following domains proposed as part of a new tool developed within the Methodological Investigation of Cochrane Reviews of Complex Interventions (MICCI) project (Simon Lewin, personal communication): (1) number of discrete, active components included in the intervention compared with the control (or usual care); (2) number of behaviors or actions of intervention recipients or participants to which the intervention is directed; (3) number of organizational levels targeted by the intervention; (4) degree of flexibility or tailoring permitted across sites or individual intervention implementation/application; (5) the level of skill required by those delivering the intervention; and (6) the level of skill required for the targeted behavior when entering the study by those receiving the intervention (consumers, professionals, planners) in order to meet the intervention’s objectives

### Types of studies to be included

#### Intervention studies

RCTs, cluster randomized controlled trials (cRCTs), NRCTs, interrupted time series studies (ITSs) and controlled before-and-after studies (CBAs) will all be included.

#### Observational studies

Uncontrolled before-and-after studies, cohort studies, case-control studies and cross-sectional studies will qualify for inclusion.

### Types of studies to be excluded

Supplementary immunization activities such as mass campaigns and school-based immunization services will be excluded.

### Types of participants

Participants will include: children under 10 years of age receiving WHO recommended vaccines through ‘routine immunization services’, pregnant women receiving tetanus toxoid (TT) vaccination according to the national immunization schedule, caregivers of children or pregnant women who are receiving the vaccines, healthcare workers administering the vaccines and health facilities or health programs providing immunization services.

### Types of interventions

Interventions for improving routine immunization coverage will be categorized in the following four groups, as implemented in a previous systematic review [9]: (1) patient-oriented or community-oriented interventions, (2) provider-oriented interventions, (3) health-system interventions, and (4) multifaceted interventions (unique combinations of any of the above).

### Outcomes

#### Primary outcome

The primary outcome will be the proportion of children who have been fully immunized by the recommended age according to the national immunization schedule, or an appropriate proxy measure such as DTP3 coverage.

#### Secondary outcomes

Secondary outcomes will be identified from included studies. These may include, but are not limited to: (1) other measures of immunization program performance as reported by the authors, for example, coverage with a specific vaccine, vaccine dropout rate, and adverse events following immunization (AEFI); (2) occurrence of vaccine preventable diseases (VPDs); (3) attitude and care-seeking behavior of caregivers towards immunization; (4) attitude and skills of healthcare workers; (5) characteristics of health facilities or health programs providing immunization services; (6) implementation of intervention; and (7) cost of intervention.

### Search strategy and sources

The following electronic databases will be searched for peer-reviewed literature: Cochrane Database of Systematic Reviews (CDSR), Cochrane Central Register of Controlled Trials (CENTRAL), MEDLINE, EMBASE published by 31 August 2013.

A comprehensive search strategy will be developed that includes terms for immunization, coverage and immunization programs; as well as terms for children. The search strategy will be adapted to suit each individual database using applicable controlled vocabulary (see Additional file 1 for the proposed MEDLINE search strategy).

### Data collection and analysis

#### Study selection

Two authors (SM and CSW) will screen titles and abstracts of studies for potential eligibility. Following this, full texts of potentially eligible studies will be retrieved. Two authors (SM and CSW) will independently apply inclusion criteria to identify relevant studies to be included in the review. Any disagreements between the two authors regarding study eligibility will be resolved by discussion and consensus, failing which a third author (ER, RvK or GDH) will arbitrate. We will provide a table with characteristics of included studies, and another of excluded studies with reasons for their exclusion, in the final review.

### Data extraction

A data extraction form will be developed by consultation and consensus among all authors. Two authors (SM and CSW) will independently extract data and assess risk of bias in included studies, compare their results, and resolve any discrepancies by discussion and consensus. We plan to analyze the data using Review Manager (RevMan).

### Assessment of risk of bias

The quality of studies will be assessed using the modified GATE tool for experimental studies and the modified GATE tool for observational studies. Details of both of these tools are provided in the updated NICE Public Health Guidance manual for 2013 [11].

Two authors (SM and CSW) will apply the inclusion criteria, and any disagreements will be resolved by discussion and consensus; failing which we will consult a third author (ER, RvK or GDH).

### Measures of treatment effect and data synthesis

We will express the result of each study as a risk ratio with its corresponding 95% confidence intervals for dichotomous data, or mean difference with its standard deviation for continuous data. We will conduct meta-analysis for the same type of participants, interventions, study designs, and outcome measures where homogeneity of data allows. We will use the random-effects model as the default procedure for meta-analyses due to anticipated heterogeneity, even if the latter is not statistically significant. If meta-analysis is not feasible due to significant statistical heterogeneity, we will explore the use of harvest plots. Harvest plots are a novel method for synthesizing evidence about the differential effects of heterogeneous and complex interventions, allowing review authors to maximize the learning potential derived from the studies included, to tailor the characteristics of studies that are most relevant within a particular body of evidence, and to aid in visualizing the results [12].

### Assessment of heterogeneity

We will assess statistical heterogeneity using the χ^2^ test of homogeneity and quantify it using the I^2^ test statistic. We will describe heterogeneity as high if the I^2^ test statistic is greater than 50%, and will consider it statistically significant if the *P* value for the χ^2^ test is ≤0.1. If studies are found to be homogeneous (that is *P* value for the χ^2^ test is >0.1), results will be pooled by random effects meta-analysis, stratified by study design. Subgroup analyses will be conducted where possible, with subgroups defined by continent (Africa, Asia, Europe, Latin America and the Caribbean), setting (for example, urban vs rural), and vaccine delivery strategies. We will also tabulate heterogeneity in context and implementation between studies, using a new tool developed as part of the EU-funded INTEGRATE-HTA project (Lisa Pfadenhauer, personal communication).

### Assessment of reporting bias

A funnel plot will be used to investigate the risk of publication bias by intervention type. The funnel plot will be visually examined for asymmetry. Provided 10 or more studies are included in the analysis for each intervention type, we will use the Beggs-Egger test to assess for funnel plot asymmetry.

### Sensitivity analyses

If there is sufficient data, we will conduct sensitivity analyses to assess the effects of missing data, study design, and risk of bias on our primary meta-analyses. When we find a study with missing data, we will first perform available cases analysis; followed by sensitivity analyses according to imputations (that is, from assuming that all missing data are failures to assuming that all missing data are successes). We will also conduct sensitivity analyses to investigate the robustness of the results to study design (intervention vs correlation), method of meta-analysis (that is, random effects vs fixed effect), and risk of bias (that is, excluding studies with high risk of bias).

This protocol has not been registered with PROSPERO.

## Discussion

### Expected significance of the study

The findings of this extensive review will provide a deeper understanding of not only which interventions have been shown to work for improving vaccination coverage in LMICs, but provide insight on how they work, why they work, and for whom these interventions work. The review will allow us to document evidence across a broad range of intervention types and also distinguish between those that are well supported by evidence (to direct policy recommendations) and those that are not well supported (to direct research agenda). In addition, understanding and documenting of study contextual factors will allow for greater understanding of external validity through detailed assessment and facilitate better replication of interventions in other locations.

With an estimated 22.4 million children reported to not have received the DTP3 vaccine in 2011, where more than 70% of these children live in 10 LMICs countries (Afghanistan, Chad, Democratic Republic of the Congo, Ethiopia, India, Indonesia, Nigeria, Pakistan, Philippines and South Africa), identifying effective interventions for improving vaccination coverage in LMICs remains key in efforts to achieve universal childhood immunization [4].

## Competing interests

The authors declare that they have no competing interests.

## Authors’ contributions

SM, ER and CSW contributed to the conception and design of this review, and will be involved in data collection and analysis. SM will analyze data with input from all authors [SM, ER, CSW, RvK, GH], and all authors will participate in the interpretation of results. All authors were involved in the drafting of this protocol and have given their approval for publication.

## Supplementary Material

Additional file 1 **MEDLINE search (adapted from Oyo-Ita *et al.*** [9]**).** Click here for file
