# Supplementary material for: Understanding interventions for improving routine immunization coverage in children in low- and middle-income countries: a systematic review protocol
Source: Syst Rev. 2013 Nov 21;2:106. doi: 10.1186/2046-4053-2-106 (PMC3843560; doi:10.1186/2046-4053-2-106)
Supplement: Additional file 1 — MEDLINE search (adapted from Oyo-Ita et al. [9]). [file 2046-4053-2-106-S1.doc]

Additional file 1

MEDLINE search (adapted from Oyo-Ita *et al*. [9]):

| Search | Query |
| --- | --- |
| #20 | Search (#16 OR #19) |
| #19 | Search ((#13 AND #17 AND #18) NOT (animals[mh] NOT humans[mh])) |
| #16 | Search ((#14 AND #15) NOT (animals[mh] NOT humans[mh])) |
| #18 | Search (pregnant women[mh] OR pregnan*[tiab] OR pregnancy[mh] OR mothers[mh] OR mother[tiab] OR mothers[tiab] OR women[mh:noexp] OR women[tiab] OR woman[tiab] female[mh] OR female*[tiab]) |
| #17 | Search (tetanus toxoid[mh] OR tetanus toxoid[tiab] OR tetanus vaccine*[tiab] OR tetanus prophylaxis[tiab]) |
| #15 | Search (child[mh] OR child[tiab] OR children[tiab] OR infant[mh] OR infant[tiab] Or infants[tiab] OR infancy[tiab] OR newborn*[tiab] OR neonat*[tiab] OR baby[tiab] OR babies[tiab] OR kid[tiab] OR kids[tiab] OR toddler[tiab] OR toddlers[tiab] OR boy[tiab] OR boys[tiab] OR girl[tiab] OR girls[tiab] OR preschool[tiab] OR pre-school[tiab] OR kindergar*[tiab] OR paediatric*[tiab] OR pediatric*[tiab] OR peadiatric*[tiab] OR creche*[tiab] OR primary school*[tiab] OR child care[mh]) |
| #14 | Search (#7 AND #13) |
| #13 | Search (#8 OR #9 OR #10 OR #11 OR #12) |
| #7 | Search (#5 OR #6) |
| #12 | Search (Afghanistan [tiab] OR Albania [tiab] OR Algeria [tiab] OR Angola [tiab] OR Antigua [tiab] OR Barbuda [tiab] OR Argentina [tiab] OR Armenia [tiab] OR Armenian [tiab] OR Aruba [tiab] OR Azerbaijan [tiab] OR Bahrain [tiab] OR Bangladesh [tiab] OR Barbados [tiab] OR Benin [tiab] OR Byelarus [tiab] OR Byelorussian [tiab] OR Belarus [tiab] OR Belorussian [tiab] OR Belorussia [tiab] OR Belize [tiab] OR Bhutan [tiab] OR Bolivia [tiab] OR Bosnia [tiab] OR Herzegovina [tiab] OR Hercegovina [tiab] OR Botswana [tiab] OR Brazil [tiab] OR Brasil [tiab] OR Brasilia [tiab] OR Bulgaria [tiab] OR Burkina Faso [tiab] OR Burkina Fasso [tiab] OR Upper Volta [tiab] OR Burundi [tiab] OR Urundi [tiab] OR Cambodia [tiab] OR Khmer Republic [tiab] OR Kampuchea [tiab] OR Cameroon [tiab] OR Cameroons [tiab] OR Cameron [tiab] OR Camerons [tiab] OR Cape Verde [tiab] OR Central African Republic [tiab] OR Chad [tiab] OR Chile [tiab] OR China [tiab] OR Colombia [tiab] OR Comoros [tiab] OR Comoro Islands [tiab] OR Comores [tiab] OR Mayotte [tiab] OR Congo [tiab] OR Zaire [tiab] OR Costa Rica [tiab] OR Cote d'Ivoire [tiab] OR Ivory Coast [tiab] OR Croatia [tiab] OR Cuba [tiab] OR Cyprus [tiab] OR Czechoslovakia [tiab] OR Czech Republic [tiab] OR Slovakia [tiab] OR Slovak Republic [tiab] OR Djibouti [tiab] OR French Somaliland [tiab] OR Dominica [tiab] OR Dominican Republic [tiab] OR East Timor [tiab] OR East Timur [tiab] OR Timor Leste [tiab] OR Ecuador [tiab] OR Egypt [tiab] OR United Arab Republic [tiab] OR El Salvador [tiab] OR Eritrea [tiab] OR Estonia [tiab] OR Ethiopia [tiab] OR Fiji [tiab] OR Gabon [tiab] OR Gabonese Republic [tiab] OR Gambia [tiab] OR Gaza [tiab] OR Georgia Republic [tiab] OR Georgian Republic [tiab] OR Ghana [tiab] OR Gold Coast [tiab] OR Greece [tiab] OR Grenada [tiab] OR Guatemala [tiab] OR Guinea [tiab] OR Guam [tiab] OR Guiana [tiab] OR Guyana [tiab] OR Haiti [tiab] OR Honduras [tiab] OR Hungary [tiab] OR India [tiab] OR Maldives [tiab] OR Indonesia [tiab] OR Iran [tiab] OR Iraq [tiab] OR Isle of Man [tiab] OR Jamaica [tiab] OR Jordan [tiab] OR Kazakhstan [tiab] OR Kazakh [tiab] OR Kenya [tiab] OR Kiribati [tiab] OR Korea [tiab] OR Kosovo [tiab] OR Kyrgyzstan [tiab] OR Kirghizia [tiab] OR Kyrgyz Republic [tiab] OR Kirghiz [tiab] OR Kirgizstan [tiab] OR Lao PDR [tiab] OR Laos [tiab] OR Latvia [tiab] OR Lebanon [tiab] OR Lesotho [tiab] OR Basutoland [tiab] OR Liberia [tiab] OR Libya [tiab] OR Lithuania [tiab] OR Macedonia [tiab] OR Madagascar [tiab] OR Malagasy Republic [tiab] OR Malaysia [tiab] OR Malaya [tiab] OR Malay [tiab] OR Sabah [tiab] OR Sarawak [tiab] OR Malawi [tiab] OR Nyasaland [tiab] OR Mali [tiab] OR Malta [tiab] OR Marshall Islands [tiab] OR Mauritania [tiab] OR Mauritius [tiab] OR Agalega Islands [tiab] OR Mexico [tiab] OR Micronesia [tiab] OR Middle East [tiab] OR Moldova [tiab] OR Moldovia [tiab] OR Moldovian [tiab] OR Mongolia [tiab] OR Montenegro [tiab] OR Morocco [tiab] OR Ifni [tiab] OR Mozambique [tiab] OR Myanmar [tiab] OR Myanma [tiab] OR Burma [tiab] OR Namibia [tiab] OR Nepal [tiab] OR Netherlands Antilles [tiab] OR New Caledonia [tiab] OR Nicaragua [tiab] OR Niger [tiab] OR Nigeria [tiab] OR Northern Mariana Islands [tiab] OR Oman [tiab] OR Muscat [tiab] OR Pakistan [tiab] OR Palau [tiab] OR Palestine [tiab] OR Panama [tiab] OR Paraguay [tiab] OR Peru [tiab] OR Philippines [tiab] OR Philipines [tiab] OR Phillipines [tiab] OR Phillippines [tiab] OR Poland [tiab] OR Portugal [tiab] OR Puerto Rico [tiab] OR Romania [tiab] OR Rumania [tiab] OR Roumania [tiab] OR Russia [tiab] OR Russian [tiab] OR Rwanda [tiab] OR Ruanda [tiab] OR Saint Kitts [tiab] OR St Kitts [tiab] OR Nevis [tiab] OR Saint Lucia [tiab] OR St Lucia [tiab] OR Saint Vincent [tiab] OR St Vincent [tiab] OR Grenadines [tiab] OR Samoa [tiab] OR Samoan Islands [tiab] OR Navigator Island [tiab] OR Navigator Islands [tiab] OR Sao Tome [tiab] OR Saudi Arabia [tiab] OR Senegal [tiab] OR Serbia [tiab] OR Montenegro [tiab] OR Seychelles [tiab] OR Sierra Leone [tiab] OR Slovenia [tiab] OR Sri Lanka [tiab] OR Ceylon [tiab] OR Solomon Islands [tiab] OR Somalia [tiab] OR Sudan [tiab] OR Suriname [tiab] OR Surinam [tiab] OR Swaziland [tiab] OR Syria [tiab] OR Tajikistan [tiab] OR Tadzhikistan [tiab] OR Tadjikistan [tiab] OR Tadzhik [tiab] OR Tanzania [tiab] OR Thailand [tiab] OR Togo [tiab] OR Togolese Republic [tiab] OR Tonga [tiab] OR Trinidad [tiab] OR Tobago [tiab] OR Tunisia [tiab] OR Turkey [tiab] OR Turkmenistan [tiab] OR Turkmen [tiab] OR Uganda [tiab] OR Ukraine [tiab] OR Uruguay [tiab] OR USSR [tiab] OR Soviet Union [tiab] OR Union of Soviet Socialist Republics [tiab] OR Uzbekistan [tiab] OR Uzbek [tiab] OR Vanuatu [tiab] OR New Hebrides [tiab] OR Venezuela [tiab] OR Vietnam [tiab] OR Viet Nam [tiab] OR West Bank [tiab] OR Yemen [tiab] OR Yugoslavia [tiab] OR Zambia [tiab] OR Zimbabwe [tiab] OR Rhodesia) |
| #10 | Search (developing[tiab] OR less* developed[tiab] OR least developed[tiab] OR under developed[tiab] OR underdeveloped[tiab] OR third world[tiab] OR middle income[tiab] OR low income[tiab] OR lower income[tiab] OR lowest income[tiab] OR underserved[tiab] OR under served[tiab] OR deprived[tiab] OR poor*[tiab]) AND (countr*[tiab] OR nation*[tiab] OR population*[tiab] OR state*[tiab] OR world[tiab] OR economy[tiab] OR economies[tiab]) |
| #11 | Search developing countries[mh] |
| #9 | Search (lmic[tiab] OR lmics[tiab] OR lami countr*[tiab] OR “low and middle income countries”[tiab]) |
| #8 | Search (Africa[mh] OR Asia[tiab] OR Caribbean region [tiab] OR South America [tiab] OR Latin America [tiab] OR Central America[tiab) |
| #6 | Search (measles vaccine[mh] OR mumps vaccine[mh] OR rubella vaccine[mh] OR measles-mumps-rubella vaccine[mh] OR poliovirus vaccines[mh] OR poliovirus vaccine, inactivated[mh] OR poliovirus vaccine, oral[mh] OR tuberculosis vaccines[mh] OR bcg vaccine[mh] OR viral hepatitis vaccines[mh] OR hepatitis b vaccines[mh] OR haemophilus vaccines[mh] OR meningococcal vaccines[mh] OR yellow fever vaccine[mh] OR rotavirus vaccines[mh] OR japanese encephalitis vaccine[mh] OR papillomavirus vaccines[mh] OR tetanus toxoid[mh] OR diptheria toxoid[mh] OR diphtheria-tetanus-acellular pertussis vaccines[mh] OR diphtheria-tetanus-pertussis vaccine[mh] OR diphtheria-tetanus vaccine[mh]) |
| #5 | Search (#3 AND #4) |
| #4 | Search (tetanus[mh] OR tetanus[tiab] OR diphtheria[mh] OR diphtheria[tiab] OR measles[mh:noexp] OR measles[tiab] OR rubeola[tiab] OR mumps[mh] OR mumps[tiab] OR epidemic parotitis[tiab] OR rubella[mh:noexp] OR rubella[tiab] OR rubellas[tiab] OR whooping cough[mh] OR whooping cough[tiab] OR pertussis[tiab] OR poliomyelitis[mh] OR polio[tiab] OR poliomyelitis[tiab] OR polios[tiab] OR infantile paralysis[tiab] OR mmr[tiab] OR tuberculosis[mh:noexp] OR tuberculosis[tiab] OR tuberculoses[tiab] OR tuberculosis, pulmonary[mh] OR mycobacterium tuberculosis[mh] OR bcg [tiab] OR calmette*[tiab] OR hepatitis b[mh] OR hepatitis b[tiab] OR viral hepatitis[tiab] OR haemophilus influenzae[mh] OR haemophilus influenza*[tiab] OR hemophilus influenza*[tiab] OR yellow fever[mh] OR yellow fever[tiab] OR meningococcal infections[mh] OR meningococcal[tiab] OR rotavirus[mh] OR rotavirus[tiab] OR encephalitis, japanese[mh] OR japanese encephalitis[tiab] OR papillomavirus[tiab] OR hpv[tiab] OR triple[tiab]) |
| #3 | Search (#1 OR #2) |
| #2 | Search (immunis*[tiab] or immuniz*[tiab] or immunotherapy[tiab] or vaccin*[tiab] or revaccinat*[tiab]) |
| #1 | Search (immunization[mh] OR immunization schedule[mh] OR immunization, secondary[mh] OR immunization programs[mh] OR vaccination[mh] OR immunotherapy, active[mh]) |
